# Improved survival and neurological outcomes with lysophosphatidylcholine supplementation in severe cardiac arrest model

**DOI:** 10.3389/fphar.2025.1587776

**Published:** 2025-08-21

**Authors:** Muhammad Shoaib, Mitsuaki Nishikimi, Rishabh C. Choudhary, Tsukasa Yagi, Kei Hayashida, Tai Yin, Blanca B. Espin, Lance B. Becker, Junhwan Kim

**Affiliations:** ^1^ Laboratory for Critical Care Physiology, The Feinstein Institutes for Medical Research, Manhasset, NY, United States; ^2^ Donald and Barbara Zucker School of Medicine at Hofstra/Northwell, Hempstead, NY, United States; ^3^ Department of Emergency Medicine, North Shore University Hospital, Manhasset, NY, United States

**Keywords:** cardiac arrest, lysophosphatidylcholine, ischemia-reperfusion injury, mass spectrometry, outcomes

## Abstract

Cardiac arrest (CA) results in a loss of blood circulation, leading to whole-body ischemia-reperfusion injuries. A deficiency in plasma lysophosphatidylcholine (LPC) levels has been observed in both human patients and rat models and is implicated in organ dysfunction following CA. Building on previous findings from a mild injury model, this study explored the therapeutic potential of LPC supplementation in a severe 12-min rat CA model. The study also compared the effects of combining multiple LPC species with individual species to better determine the most effective strategy and enhance the translational potential of LPC therapy for CA treatment. We found that LPC supplementation, particularly with LPC(18:1) alone and in combination with other LPC species, significantly improved 72-h survival rates. However, combination therapy offered superior protection compared to single LPC species, as assessed by modified neurological deficit score (mNDS). Additionally, combination therapy reduced troponin I levels, indicating cardioprotection, and facilitated the return of the N10 peak in somatosensory evoked potentials, suggesting preserved nervous system integrity. While individual LPC species offered some benefits, combination therapy yielded superior results. The significant improvements in outcomes observed in this severe model highlight the robustness of LPC therapy and its potential to treat CA patients with varying injury severities. Specifically, the use of LPC in combination emerges as a promising strategy for mitigating organ damage and enhancing recovery outcomes post-CA.

## 1 Introduction

Cardiac arrest (CA) is highly lethal, with only approximately 10% of patients surviving to hospital discharge (Virani et al., 2020). CA deprives organs of oxygen and metabolites, which are vital for their survival (Shoaib and Becker, 2022), leading to ischemic injury. Subsequent resuscitation may restore blood circulation and oxygen supply. However, the circulating blood may still lack essential nutrients vital for organ survival. While the critical role of oxygen deprivation is widely acknowledged, the significance of metabolite deprivation has received less attention. This nutrient deficiency can impede the recovery of organs from ischemic injury or even induce additional damage, contributing to the poor clinical outcomes observed, with only approximately 10% of patients surviving to leave the hospital (Virani et al., 2020).

Lysophosphatidylcholine (LPC) belongs to a family of phospholipids that contains a choline head group, a glycerol backbone, and a fatty acid tail. LPC has shown to play various roles in the body, such as acting as a signaling molecule, providing structural support for membranes, as well as serving as an energy source (Law et al., 2019; Nguyen et al., 2014). We previously showed that plasma LPC in CA patients was significantly decreased, and this decrease was strongly associated with brain injury severity and neurological outcomes (Nishikimi et al., 2022a; Nishikimi et al., 2022b). The association was replicated in our rat CA model, where we also found that supplementing plasma LPC, especially a species containing oleic acid, LPC(18:1), and docosahexaenoic acid, LPC(22:6), significantly improved rat brain function and survival after 10 min CA (Nishikimi et al., 2022a). The association with injury severity and improved outcomes with plasma LPC supplementation indicates that LPC is a critical metabolite for maintaining brain function and suggest that its deficiency may contribute to brain damage and mortality post-CA.

This study utilizes a severe rat CA model to further investigate the therapeutic benefits of LPC supplementation under more clinically relevant conditions. While our previous work demonstrated the efficacy of LPC in a less severe model, the reality is that many patients experience longer periods of CA, resulting in more profound and complex injuries. A more severe model, characterized by a prolonged period of CA, allows us to assess whether LPC supplementation can still confer benefits under these challenging circumstances. Furthermore, a more severe injury model is critical for evaluating the potential of LPC to mitigate not only the primary ischemic insult, but also the secondary cascades of injury, including inflammation and cell death, that are amplified in prolonged CA. Understanding LPC’s effects in this context is essential for translating these findings to the clinical setting where patients often present with varying degrees of injury severity.

This focused investigation in a severe injury model provides crucial pre-clinical data to support the development of LPC supplementation as a viable treatment strategy for CA survivors. By demonstrating the efficacy of LPC in mitigating the complex pathophysiology of prolonged CA, this study strengthens the rationale for human clinical trials and holds promise for improving patient outcomes across a wider range of CA-induced injury severities.

## 2 Materials and methods

### 2.1 Materials

LPC(18:0), LPC(18:1), LPC(22:6), and LPC(17:1) were purchased from Avanti Polar Lipids (Alabaster, AL). 1,2-Dipalmitoyl-sn-glycero-3-phospho-N-methylethanolamine (PME) was purchased from Santa Cruz Biotechnology (Dallas, TX). All other chemicals were purchased from Fischer or Sigma Aldrich.

### 2.2 Collection of human cardiac arrest

This study was approved by the Institutional Review Board at the Feinstein Institutes for Medical Research (protocol #17-0030). We collected plasma samples and retrospective clinical data from 46 CA patients admitted to the Emergency Department of North Shore University Hospital. All patient information was de-identified prior to analysis. Neurological outcome was assessed using the Cerebral Performance Category (CPC) score, where CPC 1-2 was defined as a good outcome and CPC 3–5 as a poor outcome (Grossestreuer et al., 2016). Detailed characteristics of the patient cohort are provided in Supplementary Table S1.

### 2.3 Animal cardiac arrest protocol

The experimental protocol was approved by the Institutional Animal Care and Use Committee of the Feinstein Institutes of Medical Research (2017–033). Adult male Sprague–Dawley rats (400–500 g, Charles River Production, Wilmington, MA) were maintained under a 12-h light/dark cycle with free access to food and water. The detailed CA procedure is provided in our previous study (Han et al., 2010; Kim et al., 2015; Kim et al. 2016; Kim et al. 2014; Kuschner et al., 2018). Rats were anesthetized with 4% isoflurane, intubated with a 14 G plastic catheter, and mechanically ventilated with oxygen containing 2% isoflurane. The left femoral artery and vein were cannulated for monitoring arterial pressure and infusing medications, respectively. Monitoring was conducted throughout all animal surgical procedures and used to follow both CA and return of spontaneous circulation (ROSC), utilizing PowerLab and LabChart software (ADInstruments, United States). The depth of anesthesia during surgical procedure was monitored by assessing heart rate, blood pressure, and absence of withdrawal response to toe pinch in all animals. Asphyxial CA was induced by switching off the ventilator and isoflurane was discontinued thereafter. CPR began with the resumption of mechanical ventilation and manual chest compressions at approximately 250 compressions per min. Thirty seconds after starting chest compressions, a 20 μg/kg bolus of epinephrine was administered, and compressions were continued. ROSC was confirmed by the achievement of a sustained arterial blood pressure of at least 60 mmHg and organized electrocardiographic activity. Typically, rats achieve ROSC within 60 s after the initiation of chest compressions. After 12 min CA and resuscitation, blood was drawn at baseline and various time points after ROSC from the femoral artery and as required by each experimental design. For survival studies, rats were weaned from the ventilator after 2 h post-ROSC and returned to their cages. Rats were block-randomized to receive either the vehicle or LPC species after 12 min CA. Rats that did not achieve ROSC were excluded from the study. Rats were euthanized by decapitation after anesthesia, in accordance with approved protocol. All aspects of animal experimentation, neurological evaluation, and LPC formulation and administration were performed in a blinded manner. The visual timeline detailing the animal protocol is provided in Supplementary Figure S1.

### 2.4 LPC preparation and administration

LPC was dissolved in 0.5% bovine serum albumin (BSA) in phosphate buffer saline (PBS; 1X, pH = 7.4) followed by five cycles of 10 s sonication. The vehicle group was simply 0.5% BSA in PBS with five cycles of 10 s sonication. Vehicle or LPC was administered within 2 min after achieving ROSC over 1 min time interval to maintain hemodynamic stability. For individual LPC species administration, 6 mg/kg dosage was used as previously determined (Nishikimi et al., 2022b). For combination LPC administration, 2 mg/kg of each LPC was combined for a total of 6 mg/kg administered in a single injection.

### 2.5 Assessment of neurological functionality post-cardiac arrest

Modified neurological deficit score (mNDS) was applied to evaluate neurological functions at 24, 48, and 72 h after ROSC for all surviving rats. The mNDS (100% representing perfect score and 0 brain dead) is composed of 5 parameters testing general appearance, cranial nerve, motor, sensory, and coordination (Supplementary Table S2) (Carrillo et al., 1998; Neumar et al., 1995). The overall performance categories (OPC) score, which is analogous to human CPC score, was assessed as shown previously (Carrillo et al., 1998; Han et al., 2010). The OPC assess the neurological and functional outcomes, ranging from 1 (good recovery) to 5 (brain death) (Supplementary Table S3).

### 2.6 SSEP measurement

A somatosensory evoke potential (SSEP) analysis was performed to assess brain function in the early phase of resuscitation up to 2 h after achieving ROSC. For SSEP measurement, rats underwent implantation of electrodes as previously published (Leanne Moon et al., 2016; Madhok et al., 2010; Shoaib et al., 2022; Xiong et al., 2010). After induction of anesthesia (4% isoflurane), rats were placed in a stereotaxic apparatus and skull was exposed. Burr holes were created in the exposed skull and 4 screw electrodes (Plastics One, Roanoke, VA, United States) were cortically implanted. There were 2 electrodes placed over the forelimb regions of the primary somatosensory cortex (0.5 mm posterior and 3.8 mm lateral to bregma) and 2 electrodes were implanted in the frontal cortex (2 mm anterior and 2 mm lateral to bregma) bilaterally (Paxinos and Watson, 2006). One electrode was placed over the parasagittal right frontal lobe as a reference/ground. The SSEP signals were recorded from the skull electrodes of forelimb primary sensory cortex following stimulation of the median nerves in both hands. Stimulation pulses (200 μs, 6 mA, 0.5 Hz) (Bazley et al., 2014; Maybhate et al., 2012) were delivered to subdermal electrodes (ADInstruments, United States) at a frequency of 0.5 Hz using an isolated stimulator (WPI, IsostimTM A320, United States). The subsequent SSEPs were recorded with the INTAN-128 channel stimulation/recording controller (INTAN Technology, United States) at a sampling rate of 30.0 kS (33.3 μS stimulation time resolution). The SSEP signals were recorded for 20 min prior to CA (baseline); started with 10 min stimulation of left hand followed by 10 min stimulation of right hand. Recording was paused during CA but resumed after ROSC, continuing in 10 min intervals until 2 h post-ROSC with alternating between right- and left-hand stimulation. The sweeps within 10 min interval were averaged and the quantitative analyses were performed on the averaged waveform for each time period and normalized to baseline values. The normalized values for all time periods were used to generate the aggregate value for each quantitative marker. The N10 peak, which is a negative deflection approximately 10 ms after stimulation and equivalent to the N20 peak in humans (Madhok et al., 2010), was used to assess brain recovery and thalamocortical interactions. The N10 amplitude was measured as the peak-to-peak amplitude between the N10 and P15 (positive deflection 15 ms after stimulation) peaks, and the N10 latency was measured as the time from stimulation to the N10 peak using a custom MATLAB algorithm (MathWorks, Natick MA). Animals with abnormal baseline waveforms (bilaterally distorted N10) were excluded from the analysis.

### 2.7 Extraction of phospholipids from plasma

Blood was collected and centrifuged at 1,000 *g* for 10 min to separate plasma. Analysis of plasma LPC was performed using an establish method (Choi et al., 2018; Kuschner et al., 2018; Nishikimi et al., 2022b). Briefly, 50 μL of frozen plasma was extracted with 750 μL of methanol. The extraction was performed in the presence of 1.7 nmol of LPC (17:1) and 0.7 nmol of PME, which served as internal standards. The mixture was vortexed for 2 min, incubated for 10–30 min at 4 °C, and centrifuged for 10 min at 14,000 x g. The supernatant (700 μL for plasma and 950 μL for tissue) was decanted and evaporated to dryness under N_2_ gas. The residue was reconstituted in a 0.2 mL of eluent A containing isopropanol: t-butyl methyl ether: aqueous ammonium formate (94 mM, pH ∼2.5) (34:17:5, v:v:v). Finally, 20 μL of the reconstitution from plasma or tissues was injected into the HPLC-MS. Various levels of biomarkers were measured in the plasma according to manufacturers’ protocols: syndecan-1 as a marker of endothelial injury (Novus Biologicals, Littleton, CO, United States), Lipocalin-2 as a marker of kidney injury (Abcam, Cambridge, United Kingdom), and cardiac troponin I as a marker of myocardial injury (Abcam, Cambridge, United Kingdom).

### 2.8 HPLC-MS analysis of LPC and PC

Analysis of LPC and PC was performed using our established method of normal-phase HPLC-MS (Kim and Hoppel, 2013; Kim et al., 2017). Normal-phase HPLC-MS was run with eluent A (isopropanol: t-butyl methyl ether: aqueous ammonium formate (94 mM, pH ∼2.5) (340:170:50, v:v:v)) and eluent B (100% methanol). The gradients used for the 35 min chromatogram will be as follows: 100% A for 18 min, 100% A to 20% A over 6 min, 20% A for 3 min, 20% A to 100% A over 1 min, and hold 100% A for 7 min. The flow rate initially was 0.30 mL/min and increased to 0.35 mL/min over 20 min and kept until the end of the run. The column temperature was 30 °C. MS and MS/MS data of LPC and PC was obtained with an LTQ XL spectrometer (Thermo Scientific, San Jose, CA) operated in the negative ion mode. The obtained data was processed using Thermo X-Calibur software (version 2.2). Retention time and MS and MS/MS data were compared to the control to confirm individual PC and LPC species. The concentration of PC and LPC were calculated as previously shown (Kim et al., 2017).

### 2.9 Statistical analysis

Data for continuous variables are presented as mean ± standard error of the mean (SEM), while categorical data are presented as counts and proportions. Statistical analyses were 1) Fisher’s exact test for categorical outcomes, 2) unpaired T-test or Mann-Whitney U test for two-group comparisons, or 3) one-way ANOVA followed by Dunnett’s multiple comparisons test for comparing multiple treatment groups to the control vehicle group. S Survival analysis was evaluated using the log-rank test, while the time to SSEP N10 peak recovery was analyzed with the Gehan-Breslow-Wilcoxon test. Statistical significance was set at P < 0.05. GraphPad Prism version 8.4 (GraphPad Software Inc., La Jolla, CA, United States) was used for statistical analyses.

## 3 Results

### 3.1 Comparison of LPC between the two rat models compared to human patients

**FIGURE 1:**
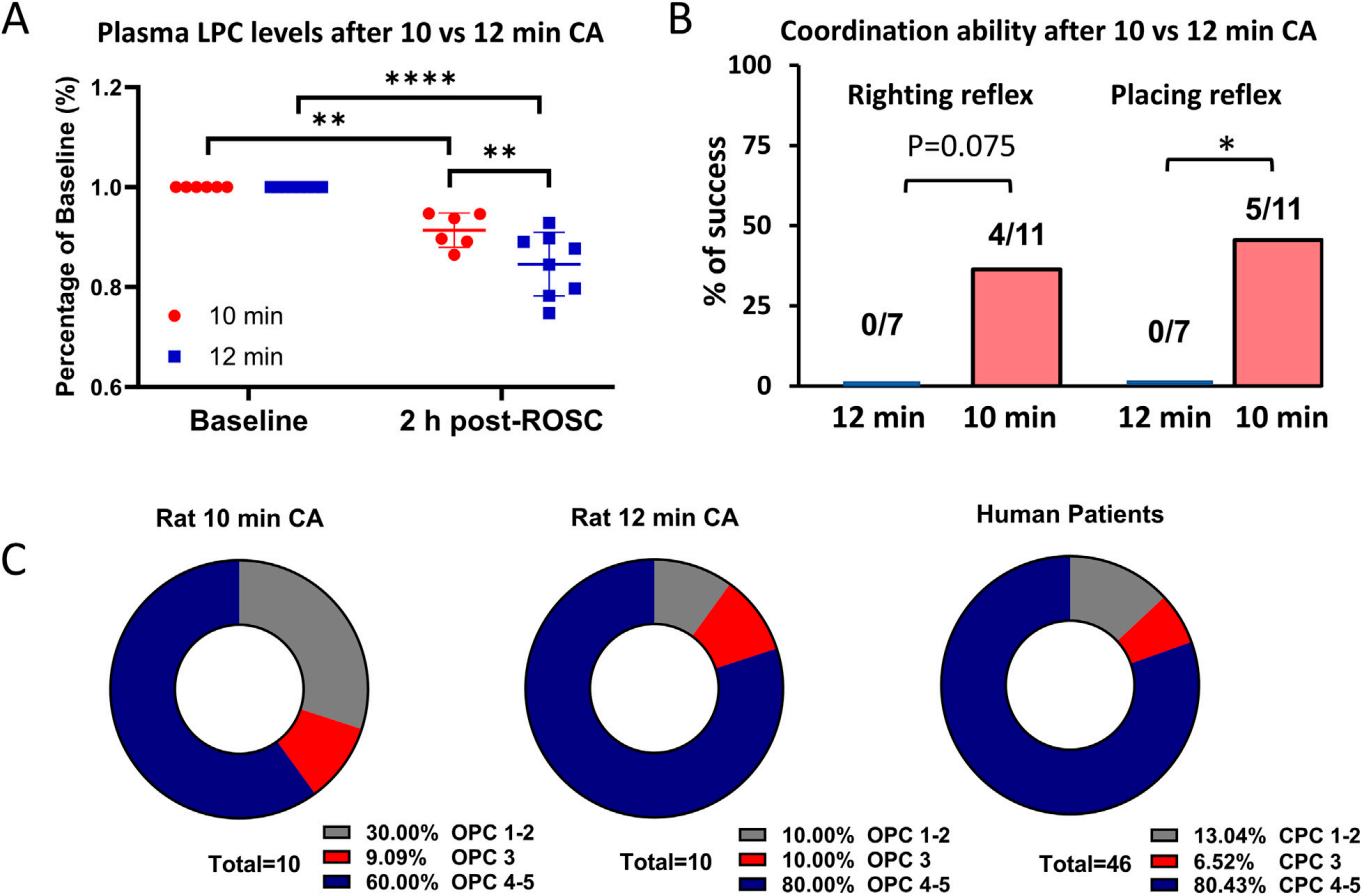
Comparison of plasma LPC levels and outcomes between after 10 min and 12 min CA. **(A)** The average plasma LPC levels are decreased by 9% at 1 h post-ROSC compared to the baseline after 10 min CA (n = 10) whereas by 17% after 12 min CA (n = 10). **(B)** Out of the surviving rats, 4/11 rats completed righting reflex test and 4/11 rats completed placing reflex test after 10 min CA. However, none of the 7 rats succeeded these challenges after 12 min CA, signifying severe neurologic damage. **(C)** The OPC scores of rats after 10 min CA seems better than rats after 12 min CA. The OPC score of rats after 12 min CA is comparable to CPC score of human patients. Student’s t-test was used to compare differences in LPC levels and Chi-square test to compare differences in righting reflex and placing reflex. Data is presented as mean ± SEM. *P < 0.05, **P < 0.01, ****P < 0.0001; CA, cardiac arrest; CPC, cerebral performance categories; CPR, Cardiopulmonary resuscitation; OPC, overall performance categories; ROSC, return of spontaneous circulation.

We first confirmed that a 12 min CA in rats induced more severe injury compared to a 10 min CA. Rats subjected to 12 min of CA exhibited significantly lower plasma LPC levels (Figure 1A), demonstrating additional 2 min ischemic time cause more biochemical alterations, resulting in a more profound depletion of this critical phospholipid (Nishikimi et al., 2022b). Consistent with this, rats surviving 12 min of CA showed significantly impaired coordination compared to those subjected to 10 min of CA. For example, while 35%–45% of rats surviving the 10-min CA completed neuronal reflex tests, none of the rats in the 12-min CA group achieved this. While 35%–45% survived rats after 10 min CA complete neuronal reflex tests whereas none of rats after 12 min CA complete these tasks (Figure 1B). We also compared plasma troponin I levels between the 10- and 12-min CA models and found no statistically significant difference (Supplementary Figure S2). This suggests that, under the current experimental conditions, blood markers may not effectively differentiate between the two groups. To provide a broader assessment of outcomes, we also utilized thee OPC scores, which are analogous to human CPC score (Carrillo et al., 1998; Han et al., 2010). We observed that rats subjected to 12 min of CA demonstrated worse performance scores, aligning more closely with the scores observed in human patients, compared to rats subjected to 10 min of CA (Figure 1C). However, we did not observe These findings collectively demonstrate that the 12 min CA model induces more severe biochemical and physiological injuries, better approximating the conditions observed in human CA patients. This provides a more relevant model for studying post-CA pathophysiology and evaluating potential therapeutic interventions.

### 3.2 Improved survival outcomes after 12 min CA with LPC supplementation

We investigated whether supplementing individual LPC species yields similar improvements in outcomes after 12 min of CA as previously demonstrated after 10 min of CA (Nishikimi et al., 2022b). The study involved two experimental phases to test each LPC species—LPC(18:0), LPC(18:1), and LPC(22:6)—as well as a combination of all three. Each experimental group consisted of 10 animals, and experiments were conducted in a strictly blinded manner with block randomization. Baseline characteristics and physiological parameters showed no differences among the groups (Supplementary Table S4).

**FIGURE 2:**
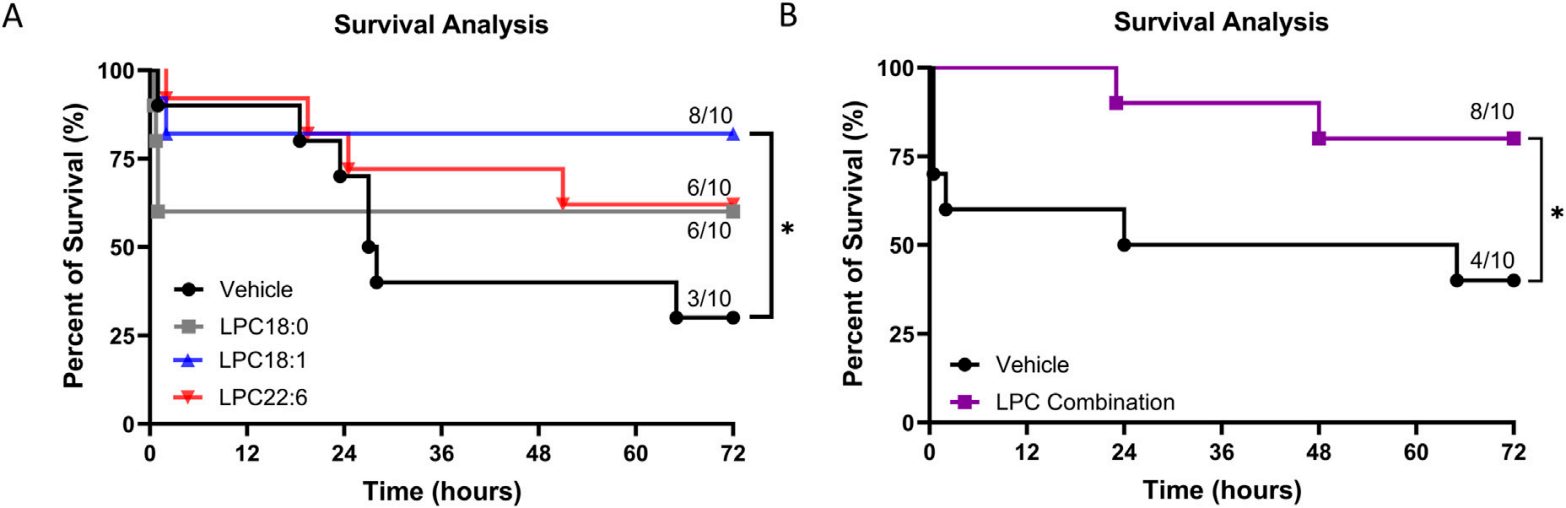
Impact of LPC supplementation on rat survival after 12 min CA. **(A)** Kaplan-Meier survival curves at 72 h for vehicle, LPC(18:0), LPC(18:1), and LPC(22:6) groups, indicating a significant increase in survival only in the LPC(18:1) group. In contrast, LPC(18:0) and LPC(22:6) show non-significant trends in survival enhancement. **(B)** Combination treatment with LPC significantly improves 72-h survival compared to the vehicle. Animals were block-randomized in a strictly blinded study design (n = 10 for each group), *P < 0.05.

Evaluation of survival among the 4 groups shows that only LPC(18:1) shows that only LPC(18:1) supplementation resulted in a statistically significant improvement in survival rates when compared to the control group. LPC(18:0) and LPC(22:6) showed positive trends in survival, but these differences did not reach statistical significance. This suggests that while all LPC species positively impacted survival, LPC(18:1) had the most pronounced effect individually (Figure 2A).

The combined LPC supplementation demonstrated a significant increase in 72-h survival compared to the control group (Figure 2B). However, while the combination treatment was beneficial, its effect on survival was similar to that observed with the most effective single treatment, LPC(18:1). This suggests that while LPC(18:1) alone is highly effective, the combination does not further enhance survival benefits beyond what LPC(18:1) achieves individually.

### 3.3 Combination LPC treatment provides better protection than LPC(18:1)

**FIGURE 3:**
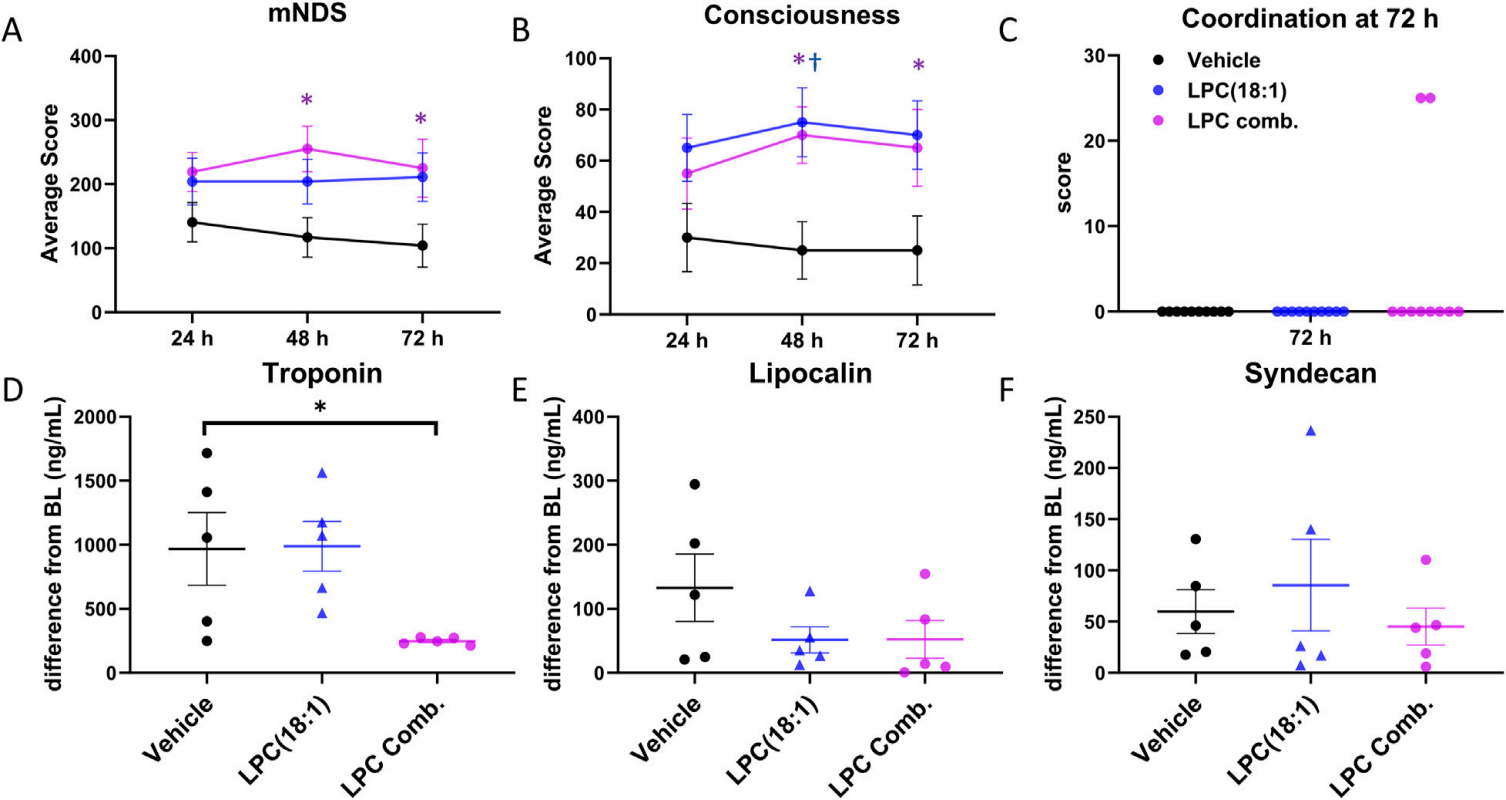
Comparing beneficial effects between LPC combination and LPC(18:1). **(A)** A significant increase in the overall mNDS was observed at 48 and 72 h in the LPC combination group, with an increasing trend noted in the LPC(18:1) group. **(B)** An enhancement in consciousness was recorded at 48 and 72 h in the LPC(18:1) group and at 48 h in the LPC combination group. **(C)** Two animals in the LPC combination group demonstrated a gain in coordination ability at 72 h, which was not observed in any other groups. **(D)** A significant decrease in plasma troponin I in the LPC combination-treated group compared to the LPC(18:1) and vehicle-treated groups suggests the supplementation attenuated cardiac injury. **(E)** Lipocalin levels showed a decreasing trend in the LPC(18:1) and LPC combination-treated groups. **(F)** Syndecan levels were similar across all three groups. Data are presented as mean ± SEM. The Student’s t-test was used for comparisons between each LPC group and the vehicle group. Non-survived animals scored 0 for mNDS and consciousness. *P < 0.05 between LPC combination and control. ^†^P < 0.05 between LPC(18:1) and control. CA - cardiac arrest; mNDS - modified neurological deficit score; black - vehicle group; blue–LPC(18:1) group; purple - LPC combination group.

To further investigate the potential benefits of combination LPC treatment, we compared its effects on neurological outcomes with those observed for LPC(18:1) alone (Figure 3). Specifically, we focused on neurological outcomes as assessed by the mNDS. For this analysis, we consolidated all vehicle-treated animals into a single control group to provide a clear baseline for comparison.

We found that the overall mNDS was significantly higher in rats treated with the LPC combination compared to vehicle-treated rats at both 48 h and 72 h (Figure 3A). While rats in the LPC(18:1) group showed an upward trend in mNDS, this did not reach statistical significance. Enhanced consciousness was observed in both the LPC combination and LPC(18:1) groups at 48 h; by 72 h, however, only the LPC(18:1) group demonstrated significantly improved consciousness (Figure 3B). Notably, the ability to demonstrate coordination—one of the most demanding tasks in the mNDS—was observed in two rats from the LPC combination group at 72 h, a response absent in the other group (Figure 3C).

We also assessed organ injury markers. Plasma troponin levels, which significantly increased at 2 h post-ROSC, were substantially reduced with LPC combination supplementation, whereas LPC(18:1) alone did not achieve this effect (Figure 3D). This suggests that the LPC combination has a beneficial effect in mitigating cardiac injury. Additionally, lipocalin levels exhibited a decreasing trend with both LPC(18:1) and LPC combination treatments, indicating potential renal protection (Figure 3E). However, syndecan levels remained consistent across all three groups, suggesting no discernible impact on endothelial injury markers (Figure 3F). These findings suggest that the LPC combination offers enhanced therapeutic effects compared to LPC(18:1) treatment alone.

**FIGURE 4:**
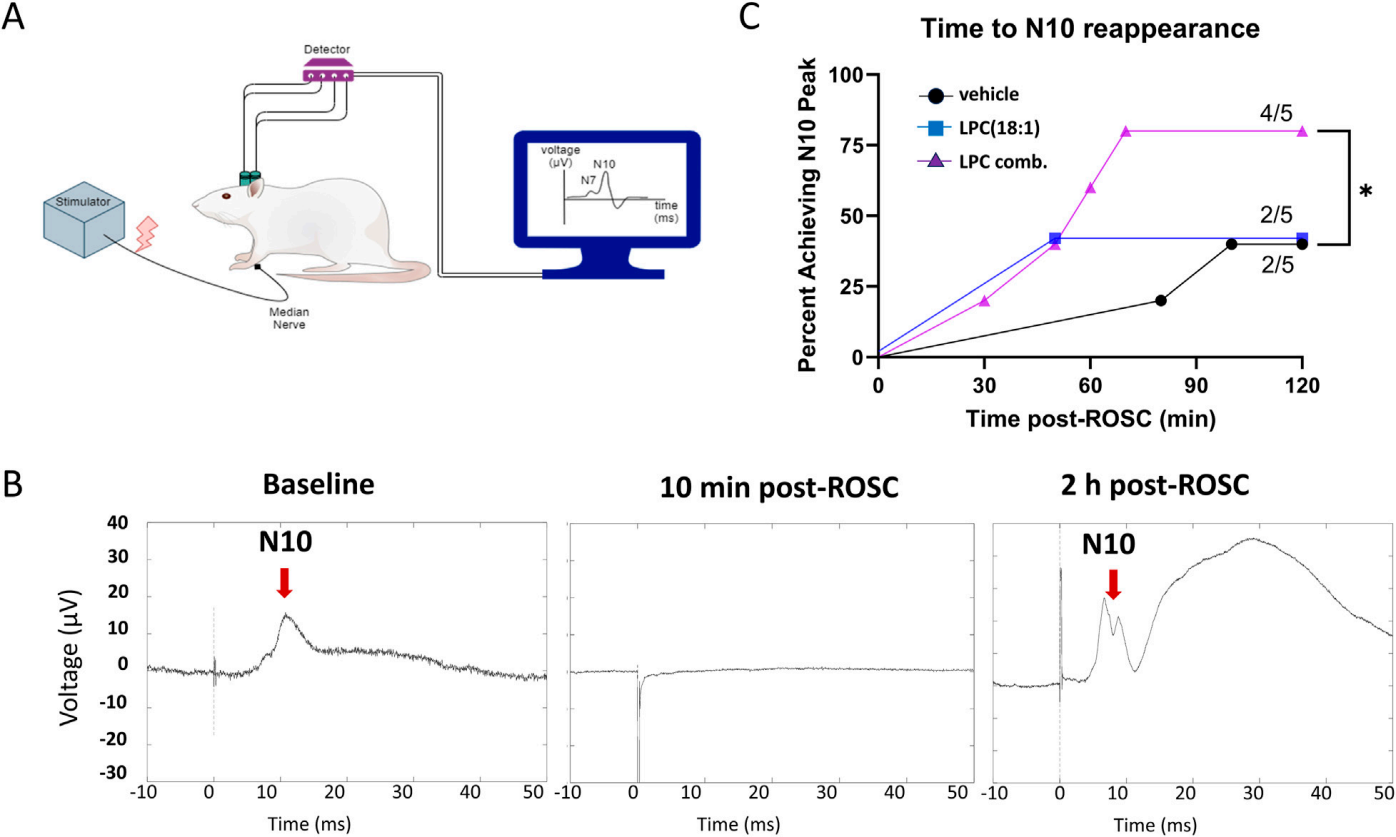
LPC combination supplementation demonstrates increased neurological protection during the early phase after ROSC as evaluated by SSEP. **(A)** Diagrammatic representation for electrode implantation for SSEP stimulation of median nerve and recording from parietal cortex to observe N7 and N10 peaks (a; n = 5). **(B)** The representative N10 (red arrows) peaks are shown at baseline, 10 min post-ROSC, and 2 h post-ROSC. **(C)** Evaluation of time to observe N10 peak post-ROSC in all groups shows that N10 peak was observed in 4/5 animals in the LPC combination group, but only 2/5 in LPC 18:1 and vehicle groups suggesting increased neuroprotection post-CA with LPC combination. The amplitude of the N10 peaks were larger than at baseline, with LPC 18:1 group showing an almost 3-fold increase in N10 amplitude, while LPC combination group was much closer to baseline levels. CA, cardiac arrest; ROSC, return of spontaneous circulation.

To achieve a more objective assessment of neuroprotective effects of LPC combination, we utilized quantitative SSEP to measure the integrity of the nervous system during the early phase of resuscitation (Figure 4). Stimulation of the median nerve bilaterally was recorded from parietal cortex to detect N10 peak (Figure 4A), which is equivalent to N20 in humans (Madhok et al., 2010). There is a loss of N10 peaks after CA and ROSC, which can slowly begin to reappear over time, signifying either return or no return of neurological activity. An N10 peak was observed in 4/5 animals in the LPC combination group, but only 2/5 in LPC 18:1 and vehicle groups (Figure 4B). Examples of the disappearance and reappearance of the N10 peaks after CA are shown in Figure 4C. Overall, our SSEP data further demonstrate that LPC combination administration improved neurological activity post-CA.

## 4 Discussion

Our study highlights the therapeutic potential of LPC supplementation in reducing organ damage and enhancing survival following severe CA in a rat model. The observed additional benefits from combining multiple LPC species indicate that each species uniquely supports cellular functions, and that their combined actions may be synergistic due to their diverse biochemical characteristics. Notably, the selected LPC species containing 18:0 (saturated), 18:1 (monounsaturated), and 22:6 (polyunsaturated) represent a spectrum of fatty acid saturation, which contributes to a broader range of biological activities. This approach offers promising avenues for further refinement and optimization, reinforcing the viability of LPC therapy.

In this study, we observed that while individual LPC species conferred 72-h survival benefits, they did not significantly improve overall mNDS (Figure 4). This contrasts with the benefits observed in a moderate CA injury rat model (Nishikimi et al., 2022b) and suggests that the injury sustained after 12 min of CA may be too extensive for single LPC species to achieve clearly observable neurofunctional recovery. Given the diverse roles of individual LPC species and the multifaceted nature of post-CA organ damage, we investigated the potential of a combination LPC treatment strategy. LPC(18:0) contains a saturated fatty acid, which serves as a major structural component of cellular membranes (Woldseth et al., 1993). LPC(18:1) includes a monounsaturated fatty acid that is uniquely abundant in the brain, influencing membrane fluidity and activating anti-inflammatory signaling pathways (Santa-María et al., 2023). LPC(22:6) is characterized by a polyunsaturated omega-3 fatty acid that acts as a source of pro-resolving mediators, supporting neuroprotection and resolving inflammation (Julliard et al., 2022).

By combining these three LPCs, we maintained the total LPC concentration as in individual treatments but used one-third the concentration (2 mg/kg) of each LPC species. This approach avoids supraphysiological levels of total LPC species, thereby minimizing the risk of off-target effects or toxicity and allowing for potential synergistic benefits. This combination therapy yielded significant improvements in both survival and mNDS. Moreover, the combination therapy reduced troponin I levels, a marker of cardiac injury, an effect not observed with the individual LPC treatments (Figure 3). These findings confirm that each LPC species leverages distinct mechanisms of action, and together, they can potentially address a broader spectrum of injury processes than any single species alone. Furthermore, these results highlight the potential for further therapeutic enhancement by incorporating additional LPC species into combination regimens.

This study has several limitations. While we observed clear cardio- and neuroprotective effects of LPC combination therapy, the precise molecular mechanisms of the synergy remain to be elucidated. Future work should dissect the interactions of individual LPC species with specific receptors and signaling pathways in relevant cell types (neurons, cardiomyocytes, endothelial cells). Additionally, we did not explore the optimal therapeutic window or alternative dosing regimens. Systematic assessment of treatment timing, duration, and LPC combinations is needed. Finally, the translatability of our findings from a rat CA model to human CA requires validation in larger animal models and clinical trials.

In conclusion, our findings provide compelling evidence for the therapeutic potential of LPC supplementation, particularly combination therapies, in improving post-CA outcomes. The observed benefits in survival, neurological function, and attenuation of cardiac injury across both 10-min and 12-min CA models, coupled with the association between LPC deficiency and injury severity, support the critical role of LPC in post-CA recovery. While further research is necessary to definitively establish causality and optimize treatment strategies, LPC supplementation represents a promising therapeutic approach with significant clinical potential.

## Data Availability

The raw data supporting the conclusions of this article will be made available by the authors, without undue reservation.
