# Supplementary material for: Improved survival and neurological outcomes with lysophosphatidylcholine supplementation in severe cardiac arrest model
Source: Front Pharmacol. 2025 Aug 21;16:1587776. doi: 10.3389/fphar.2025.1587776 (PMC12408257; doi:10.3389/fphar.2025.1587776)
Supplement: Supplementary file 1 [file Supplementaryfile1.docx]

Supplementary Material

# Supplementary Figures and Tables

**Supplementary Table S1.** Baseline characteristics of 46 post-CA patients.

| Variable | Value |
| --- | --- |
| Patient Characteristics |  |
| Age, years, median (IQR) | 76 (70–87) |
| Sex, women, n (%) | 21 (47%) |
| Comorbidities |  |
| Hypertension, n (%) | 33 (75%) |
| Diabetes mellitus, n (%) | 17 (39%) |
| Ischemic heart disease, n (%) | 22 (50%) |
| Chronic lung disease, n (%) | 6 (14%) |
| Location of arrest, n (%) |  |
| Home/Residence | 33 (75%) |
| Public place/Other | 5 (11%) |
| In-hospital | 6 (14%) |
| Presumed etiology, n (%) |  |
| Cardiac | 20 (45%) |
| Respiratory/Asphyxia | 4 (9%) |
| Other/Unknown | 19 (43%) |
| Witnessed arrest, n (%) | 30 (68%) |
| Bystander CPR performed, n (%) | 17 (38%) |
| Initial rhythm shockable, n (%) | 16 (36%) |
| Time from call to EMS arrival, min, median (IQR) | 0.65 (0.42–1.00) |
| Time to first defibrillation, min, median (IQR) | 2 (1–10) |
| Epinephrine administered pre-ROSC, n (%) | 34 (77%) |
| Number of epinephrine treatments, median (IQR) | 4 (2–5) |
| Time until ROSC, min, median (IQR) | 26.0 (11.5–42.5) |
| Survival to hospital admission, n (%) | 46 (100%) |
| Survival to hospital discharge, n (%) | 8 (17%) |

**Abbreviations:** IQR = interquartile range; ROSC = return of spontaneous circulation.

##
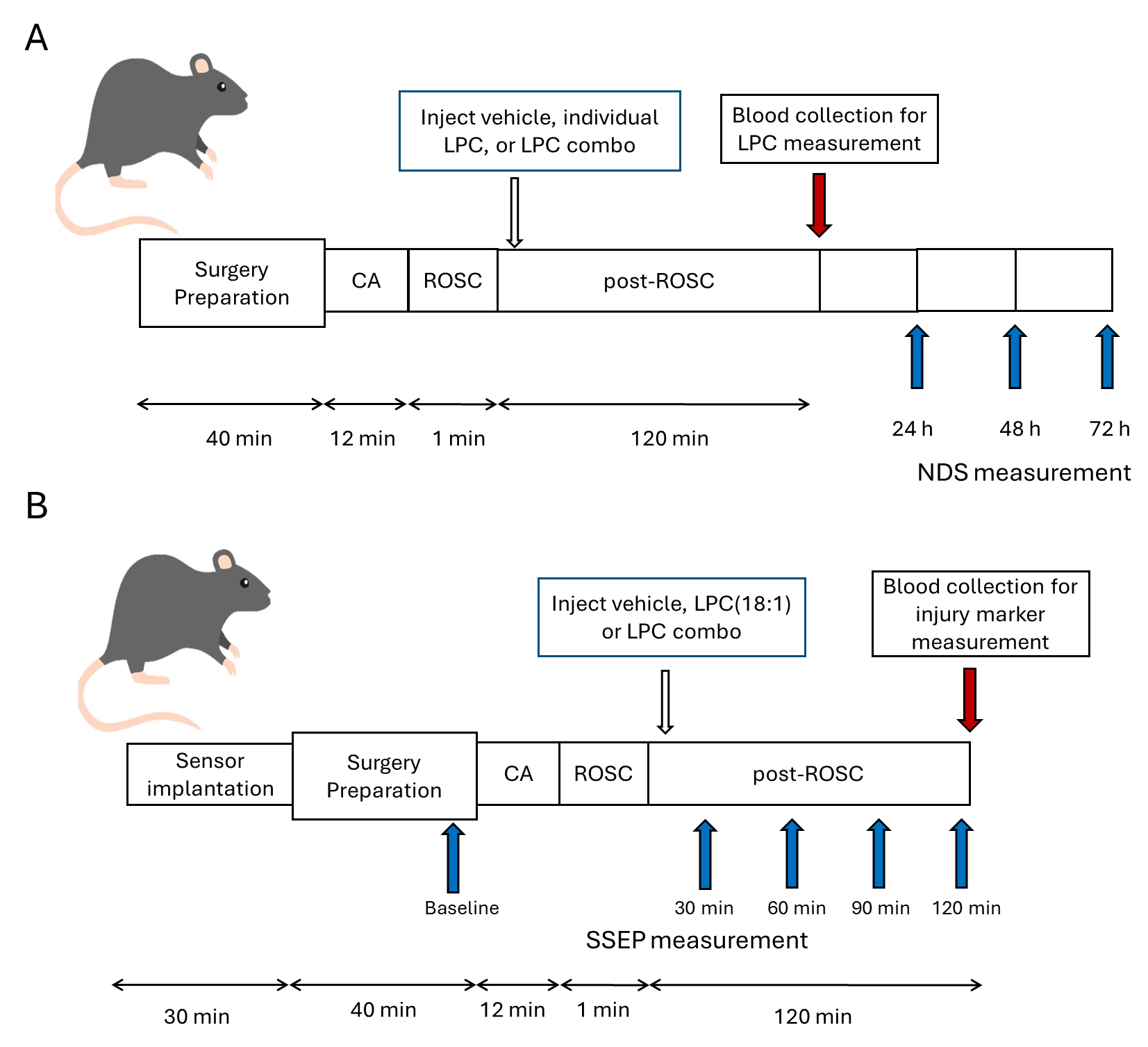


## Supplementary Figure S1. Schematic of the animal study protocol. A. Timeline illustrating the 72-hour survival study. Rats underwent 12 minutes of cardiac arrest (CA) followed by resuscitation (return of spontaneous circulation, ROSC). Immediately upon ROSC, animals were randomized to receive vehicle or one of four treatments: LPC(18:0), LPC(18:1), LPC(22:6), or a combination (n = 10 per group). Neurological deficit scores (NDS) were assessed at 24, 48, and 72 hours post-ROSC, with survival monitored throughout the 72-hour study period. Key interventions and assessments are indicated along the timeline. B. Timeline depicting the somatosensory evoked potential (SSEP) study protocol. Baseline SSEP measurements were recorded prior to CA, followed by assessments at 30, 60, 90, and 120 minutes post-ROSC. Rats were randomized to receive vehicle, LPC(18:1), or the LPC combination treatment (n=5). This protocol allowed for evaluation of early neurological recovery following treatment.

## Supplementary Table S2. Modified neurological deficit score (mNDS) for evaluation of neurological functions after cardiac arrest in rats.

|  | **Modified neurological deficit score (mNDS)** |  |  |
| --- | --- | --- | --- |
| **General** |  |  |  |
| Consciousness | Unresponsive (0), depressed (50), normal (100) |  |  |
| Respiration | Abnormal (<60 or >120) (0), normal (100) |  |  |
|  |  | Total points | /200 |
| **Cranial nerves** |  |  |  |
| Olfactory | Orient to smell: no (0), yes (20) |  |  |
| Vision | Visual stimulus startle response: no (0), yes (20) |  |  |
| Corneal reflex | Blink response to corneal stimulus: no (0), yes (20) |  |  |
| Whisker movement | Spontaneous: no (0), yes (20) |  |  |
| Hearing | Startle response to loud noise: no (0), yes (20) |  |  |
|  |  | Total points | /100 |
| **Motor** |  |  |  |
| Left forepaw | Spontaneous or withdraw from pain: no (0), yes (10) |  |  |
| Right forepaw | Spontaneous or withdraw from pain: no (0), yes (10) |  |  |
| Left hindpaw | Spontaneous or withdraw from pain: no (0), yes (10) |  |  |
| Right hindpaw | Spontaneous or withdraw from pain: no (0), yes (10) |  |  |
| Tail | Spontaneous or withdraw from pain: no (0), yes (10) |  |  |
|  |  | Total points | /50 |
| **Sensory** |  |  |  |
| Left forepaw | React to pain: no (0), yes (10) |  |  |
| Right forepaw | React to pain: no (0), yes (10) |  |  |
| Left hindpaw | React to pain: no (0), yes (10) |  |  |
| Right hindpaw | React to pain: no (0), yes (10) |  |  |
| Tail | React to pain: no (0), yes (10) |  |  |
|  |  | Total points | /50 |
| **Coordination** |  |  |  |
| Ledge traverse | no (0), yes (25) |  |  |
| Righting reflex | no (0), yes (25) |  |  |
| Placing test | no (0), yes (25) |  |  |
| Stop at table edge | no (0), yes (25) |  |  |
|  |  | Total points | /100 |
|  |  | **Total score** | **/500** |

**Supplementary Table S3.** Overall performance categories (OPC) for evaluation of outcomes after cardiac arrest in rats.

| **OPC** | **Overall Performance Categories** |
| --- | --- |
| **1** | Good overall performance. Healthy, alert, capable of normal life. Good cerebral performance plus no or only mild functional disability from noncerebral organ system abnormalities. |
| **2** | Moderate overall disability. Conscious. Sufficient cerebral function for independent activities of daily life (walking, eating, and drinking). May have hemiplegia, seizures, ataxia, dysarthria, dysphasia or permanent memory changes. |
| **3** | Several overall disability. Conscious. Dependent on others for daily support because of impaired brain function. At least limited cognition. Includes a wide range of cerebral abnormalities from ambulatory with severe memory disturbance or dementia precluding independent existence to paralytic or severe disability from noncerebral organ system dysfunction alone or both. |
| **4** | Coma, vegetative state. Not conscious. Unaware of surroundings, no cognition. No psychological interactions with environment. |
| **5** | Death. Certified brain dead or dead by traditional criteria. |

**Supplementary Figure S2. Comparison of plasma troponin I levels in rats subjected to 10-min and 12-min cardiac arrest.** No significant difference in the increase of plasma troponin I was observed between the two groups. Data are presented as mean ± SD.

**Supplementary Table S4**. Group characteristics and baseline physiological variables.

|  | **Vehicle** | **LPC 18:0** | **LPC 18:1** | **LPC 22:6** | **LPC Comb.** |
| --- | --- | --- | --- | --- | --- |
| **Rat Weights (g)** | 428.9 ± 9.0 | 430.1 ± 9.9 | 431.6 ± 8.9 | 412.9 ± 3.3 | 436.1 ± 4.7 |
| **Time to CA (min)** | 181.4 ± 5.9 | 164.4 ± 6.9 | 163.6 ± 10.1 | 181.4 ± 10.1 | 166.3 ± 6.8 |
| **Time to ROSC (min)** | 51.6 ± 6.0 | 47.9 ± 4.9 | 45.0 ± 1.0 | 45.9 ± 1.6 | 49.6 ± 2.1 |
| **Esophageal Temperature at Baseline (°C)** | 37.1 ± 0.2 | 36.4± 0.4 | 37.1 ± 0.2 | 37.3 ± 0.2 | 36.4 ± 0.4 |
| **Mean Arterial Pressure at Baseline (mmHg)** | 86.7 ± 9.6 | 84.9 ± 6.2 | 91.76 ± 3.4 | 91.5 ± 6.1 | 84.9 ± 6.2 |
| **Heart Rate at Baseline**  **(bpm)** | 288.8 ± 28.7 | 269.9 ± 15.5 | 293.3 ± 13.7 | 294.0 ± 16.7 | 269.9 ± 15.5 |
| **Systolic Pressure at Baseline (mmHg)** | 114.2 ± 11.7 | 106.7 ± 7.7 | 119.7 ± 7.0 | 120.7 ± 8.1 | 106.7 ± 7.7 |
| **Diastolic Pressure at Baseline (mmHg)** | 73.0 ± 8.7 | 73.9 ± 5.9 | 77.8 ± 4.1 | 76.9 ± 5.2 | 73.9 ± 5.9 |

^1^, one-way analysis of variance (ANOVA) followed by Tukey’s multiple comparisons test was used for the analysis. Data are presented as mean ± standard error of the mean.
